# Allergic adverse events following 2015 seasonal influenza vaccine, Victoria, Australia

**DOI:** 10.2807/1560-7917.ES.2017.22.20.30535

**Published:** 2017-05-18

**Authors:** Hazel J Clothier, Nigel Crawford, Melissa A Russell, Jim P Buttery

**Affiliations:** 1 SAEFVIC, Murdoch Childrens Research Institute, Victoria, Australia; 2 School of Population & Global Health, University of Melbourne, Victoria, Australia; 3 Department of Pediatrics, University of Melbourne, Victoria, Australia; 4 Infection and Immunity, Monash Children's Hospital & Monash Immunisation, Monash Health, Victoria, Australia; 5 Department of Paediatrics, Monash University, The Ritchie Centre, Hudson Institute, Victoria, Australia; 6 School of Population Health and Preventive Medicine, Monash University, Victoria, Australia

**Keywords:** Influenza, surveillance, vaccines and immunisation, adverse events

## Abstract

Australia was alerted to a possible increase in allergy-related adverse events following immunisation (AEFI) with 2015 seasonal trivalent influenza vaccines (TIV) by the Victorian state vaccine safety service, SAEFVIC. We describe SAEFVIC’s initial investigation and upon conclusion of the 2015 influenza vaccination programme, to define the signal event and implications for vaccine programmes. Allergy-related AEFI were defined as anaphylaxis, angioedema, urticaria or generalised allergic reaction. Investigations compared 2015 TIV AEFI reports to previous years as proportions and reporting risk (RR) per 100,000, stratified by influenza vaccine brand. The initial investigation showed an increased proportion of allergy-related AEFI compared with 2014 (25% vs 12%), predominantly in adults, with insufficient clinical severity to alter the programme risk-benefit. While overall TIV AEFI RR in 2015 was similar to previous years (RR: 1.07, 95% confidence interval (CI): 0.88–1.29), we identified a near-doubling RR for allergy-related AEFI in 2015 (RR: 1.78, 95% CI: 1.14­– 2.80) from 2011 to 2014 with no difference by vaccine brand or severity increase identified. This increase in generalised allergy-related AEFI, across all used vaccine brands, supports evidence of variable reactogenicity arising from influenza vaccine strain variations. This investigation underlines the importance of effective seasonal influenza vaccine pharmacovigilance.

## Introduction

The Australian southern hemisphere seasonal influenza vaccination programme typically runs from March to September, with influenza vaccine funded through the National Immunisation Programme (NIP) for healthcare workers, adults > 65 years of age and individuals over 6 months of age with special risk conditions [1]. Adverse events following immunisation (AEFI) are reported to the relevant jurisdictional surveillance system, which in the Australian state of Victoria is via voluntary reporting to the Victoria vaccine safety service, SAEFVIC. SAEFVIC was established in 2007 and comprises a passive surveillance system coupled with clinical services [2]. AEFI reports to SAEFVIC are received primarily as unsolicited reports from immunisation providers or healthcare workers, with direct reporting from vaccinees or their guardians accounting for approximately one fifth of reports [3]. Immunisation nurses review all reports and provide follow-up, including referral for specialist clinical consultation as required.

Influenza vaccines have been subject to additional safety surveillance monitoring in Australia since an episode of increased reactivity of one seasonal trivalent influenza vaccine (TIV) brand occurred in 2010, causing high fever and febrile seizures in children aged under 5 years [4,5].

In week 2 of the 2015 TIV Influenza vaccine programme, SAEFVIC nurses receiving AEFI reports were alerted to a possible increase in allergy-related AEFI with TIV. This possible signal was reported to the national regulatory authority, the Therapeutic Goods Administration (TGA) [6]. This paper describes SAEFVIC’s investigation initially and upon conclusion of the 2015 influenza vaccination season to define the signal event and provide guidance for future vaccine pharmacovigilance.

## Methods

Reported AEFI with influenza vaccines were extracted by date reported. Data included demographic details as well as information on vaccine administered, reactions experienced and clinical consultations. Reports of drug administration errors not resulting in AEFI were excluded. Allergy-related AEFI were clinically defined as cases of confirmed anaphylaxis, angioedema, urticaria or generalised non-specific allergic reaction. Reports of anaphylaxis were confirmed according to Brighton Collaboration case definitions [7]. All other AEFI reported, including less defined symptom descriptions of itchiness, pruritus or rash with no further description, were categorised as ‘Other’. Analyses were conducted using Excel (Microsoft Corporation, Redmond, WA, US) and STATA 13, StataCorp, Texas, US). Research Ethics and Governance of the Royal Childrens Hospital, Victoria granted approval for this study (DA017–2015–07).

### Initial investigation

The initial investigation compared the proportion of allergy-related AEFI reported to SAEFVIC with any influenza containing vaccine between 1 January and 3 May 2015 with proportions to similarly categorised data received for the whole of 2014, as convenience comparison data. In addition the Australian regulatory authority, TGA, was notified and publicly accessible data from the national Database of Adverse Event Notifications (DAEN) [8] were accessed for any allergy-related AEFI with TIV with the aim to provide insight on the national distribution of the potential signal.

Monitoring of the proportion of allergy-related AEFI was conducted through the remainder of the influenza season (April–October), with comparison to 2014 using the chi-squared test and alongside individual clinical review of serious AEFI, including anaphylaxis, as a determinant of clinical severity.

### Signal investigation

On conclusion of the southern hemisphere seasonal TIV programme on 31 October 2015, additional analysis of all SAEFVIC TIV AEFI reports for the previous eight seasons (since system commencement), 2008–2015, was conducted to define the signal event.

Data included in this analysis were restricted to the TIV brands used in the NIP in Victoria (Fluarix, GlaxoSmithKline; Fluvax, BioCSL; and Vaxigrip/Vaxigrip Junior, Sanofi Pasteur) and for which dose distribution data were available. AEFI reports received in 2015 were analysed by allergic AEFI categories, vaccinee age, sex, time to symptom onset and TIV brand. The frequency of reporting was assessed by calendar week and also realigned by week of seasonal influenza AEFI reporting commencement, as this varies each year depending on the NIP.

AEFI reporting risks (RR) were calculated using the number of vaccine doses distributed as the denominator. Doses distributed data were provided on request from the Victorian Department of Health and Human Services for 2011–2015 [9]. RR ratios were calculated, using AEFI reports received per 100,000 doses distributed.

Anaphylaxis AEFI were compared as proportion of all TIV-AEFI and as proportion of allergy-related AEFI as an indicator of clinical severity, with comparisons to combined data 2011–2014 using two-sample test of proportions with 95% confidence intervals.

## Results

### Initial investigation

At 3 May 2015, 11 (25%) of the 44 TIV AEFI reports received were allergy-related; this was an increase from 15 (12%) of 128 reports received by SAEFVIC throughout 2014 (RR: 2.13, 95% confidence interval (CI): 1.00 to 4.56).

The national DAEN database was accessed but was unable to inform the investigation as data were available only to end of January 2015, before season commencement. Personal communication with other jurisdictions and review by the TGA and the Advisory Committee on the Safety of Vaccines (ACSOV) acknowledged the potential signal in Victoria, but agreed there was insufficient evidence of clinical severity to alter the risk-benefit of the ongoing influenza vaccination programme [10].

**Figure 1:**
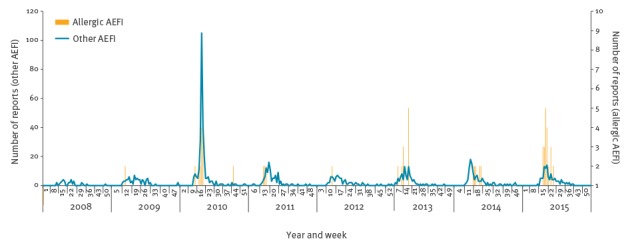
Reports of trivalent influenza vaccine adverse events following immunisation by allergy-related category and week reported, Victoria, Australia, 2008–2015

Ongoing monitoring remained suggestive of above-expected reporting of allergy-related AEFI, although frequency of reporting decreased towards the end of May (Figure 1).

AEFI: adverse events following immunisation.

The peak in 2010 corresponds to febrile-seizure signal event and increased reporting of febrile AEFI.

No increase was observed at any time during the 2015 season in the number or proportion of anaphylaxis cases, which peaked at six cases (21% [6/28] of allergy-related AEFI), when compared with four cases (27% [4/15]) reported in 2014 (p = 0.70).

### End of season signal investigation

For the 2015 season SAEFVIC received 140 reports of AEFI with TIV, of which 28 (20%) were allergy-related. Vaccinee age ranged from 0 to 79 years (mean: 36 years), with all allergy-related cases aged over 5 years (6/28 aged 6–17 years and 22/28 aged ≥18 years, mean: 39 years). While females comprised the majority of reports (104/140, 74%), the proportion reporting allergy-related AEFI was similar in both males (7/36, 19%) and females (21/104, 20%) (p = 0.92; 95% CI: -0.14 to 0.16). Three-quarters (76%) of those experiencing allergy-related AEFI reported symptom onset within one-hour of vaccination and all within 10 hours. The proportion of allergy-related AEFI with TIV of 20% (28/140) in 2015 was higher than in any previous year and significantly higher than the 12% (121/1,010) for all years 2008–2015 combined (p = 0.008; 95% CI 0.01 to 0.15) (Table 1).

**Table 1:** Trivalent influenza vaccine adverse events following immunisation reports, by category and comparison of proportion and adverse events following immunisation reports per 100,000 doses distributed, Victoria, Australia 2008–2015 (n = 1,010)

Year	All reports	Allergy reports n	Allergy reports % (95% CI)	Doses distributed	AEFI per 100,000 doses distributed	Allergic AEFI per 100,000 doses distributed
**2015**	140	28	20 (14–28)	1,186,417	11.8	**2.4**
**2014**	128	15	12 (7–19)	1,097,024	11.7	**1.4**
**2013**	116	17	15 (9–22)	1,095,217	10.6	**1.6**
**2012**	90	9	10 (5–18)	966,393	9.3	**0.9**
**2011**	119	13	11 (6–18)	932,246	12.8	**1.4**
**2010**	293	22	8 (5–11)	NA	NA	**NA**
**2009**	89	14	16 (9–25)	NA	NA	**NA**
**2008**	35	3	9 (2–23)	NA	NA	**NA**
**2008–2015**	1,010	121	12% (10–14)	NA	NA	**NA**
**2011–2014**	453	54	12% (9–15)	4,090,880	11.1	**1.3**

AEFI: adverse events following immunisation; CI: confidence interval; NA: not available.

**Figure 2:**
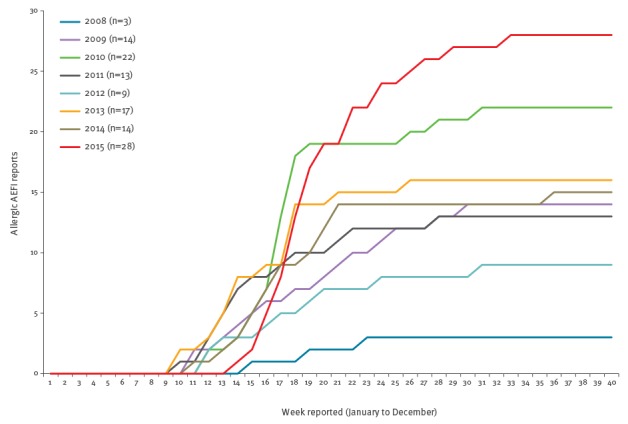
Cumulative reports of allergy-related adverse events following immunisation with trivalent influenza vaccine, by week reported, Victoria, 2008–2015 (n = 120)

**Figure 3:**
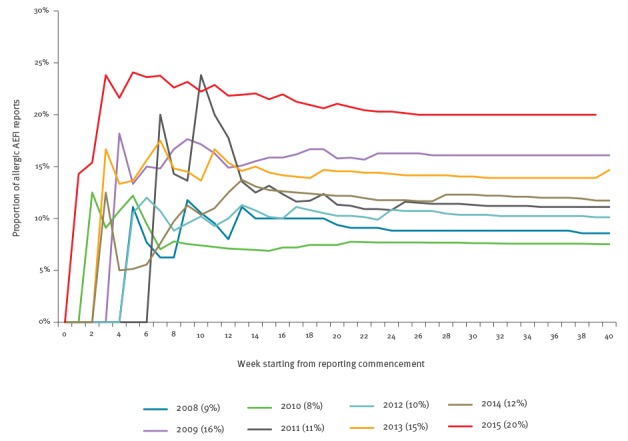
Proportion of allergy-related adverse events following immunisation (AEFI) with trivalent influenza vaccines, by week from reporting commencement, Victoria, 2008–2015 (n = 1,010 total AEFI)

The seasonal reporting pattern of overall TIV AEFI reporting in 2015 was similar to that seen in previous years (Figure 1). However, comparison of the number of allergy-related AEFI reported with previous years demonstrated the early steep rise and increased cumulative magnitude in reports (Figure 2), which was more clearly evident when realigned by weekly seasonal influenza AEFI reporting commenced in each annual period (Figure 3).

AEFI: adverse events following immunisation.

The numbers in the legend represent cumulative reports per year.

AEFI: adverse events following immunisation.

The y axis shows the proportion of allergy-related AEFI at the end of seasonal vaccination campaign.

Final analysis at 2015 seasons end found the overall 2015 TIV AEFI RR of 11.8 reports per 100,000 vaccine doses distributed was no different to the RR of 11.1 per 100,000 for the four years 2011–2014 combined (RR: 1.07, 95% CI: 0.88 to 1.29) (Table 1). However when comparing allergy-related AEFI with TIV, the RR in 2015 of 2.4 per 100,000 vaccine doses distributed was nearly double that of the combined risk of 1.3 in 2011–2014, (RR: 1.78, 95% CI: 1.14 to 2.80).

The observed variation in AEFI reports per 100,000 doses distributed by vaccine brands in 2015 was not significant either comparing individual brands, or when comparing Fluarix to the combined BioCSL Fluvax and Vaxigrip RR (RR: 1.89, 95% CI: 0.79 to 4.48) (Table 2). Anaphylaxis AEFI reports in 2015 did not differ from 2011 to 2014 combined data as a proportion of all AEFI (6/140 (4.2%) vs 9/453 (2.0%); p = 0.13) or proportion of allergy-related AEFI (6/28 (2.1%) vs 9/54, (1.7%); p = 0.60).

**Table 2:** Reports of trivalent influenza vaccine adverse events following immunisation, by symptom and reports per 100,000 doses distributed, by influenza vaccine brand, Victoria, Australia 2015 (n = 140)

Brand	All reports	Allergic reports (n)	Allergic reports (%)	Anaphylaxis	Angiodema	Urticaria	Allergic reaction generalised	Vaccine doses distributed	AEFI reports per 100,000 doses distributed	Allergic AEFI reports per 100,000 doses distributed	Comparison of Fluarix to other brands RR (95% CI)
**Fluarix^a^**	36	7	19%	2	1	1	3	212,605	16.9	3.3	Reference
**bioCSL Fluvax**	44	8	18%	2	1	4	1	586,250	7.5	1.4	2.41 (0.90–6.44)
**Vaxigrip** **(including Jnr)**	43	9	21%	2	1	5	1	387,562	11.1	2.3	1.42 (0.53–3.79)
**Brand unknown**	17	4	24%	0	1	1	2	NA	NA	NA	NA
**Total**	**140**	**28**	**20%**	**6**	**4**	**11**	**7**	**1,186,417**	**11.8**	**2.4**	

AEFI: adverse events following immunisation; NA: not available.

^a^ Fluarix was the National Immunisation Programme- funded vaccine for Victoria.

## Discussion

On conclusion of the 2015 southern hemisphere TIV/influenza season our study found a near-doubling of annual generalised allergy-related AEFI compared with the 4 previous years in this investigation of passive surveillance reports, with no evidence of correlation with any specific vaccine brand or allergic symptom, nor a significant increase in anaphylaxis. Further studies would be required to confirm that the signal was more than a spurious increase in reporting, however we were not aware of any publicity or event that may have stimulated reporting. Our study demonstrated that the longitudinal data availability, combined clinical and epidemiological services of SAEFVIC were well placed to identify and conduct a rapid investigation of a possible signal event early in the influenza vaccination campaign and facilitate evidence-based decision making by the Australian national regulatory authority.

However, vaccine pharmacovigilance in Australia is limited by wide variation in AEFI surveillance systems across Australian health jurisdictions [11]. Data are not consistent in format, categorisation or method of analyses until final collation of jurisdictional reporting and classification according to standardised MedDRA terminology is completed by the TGA. National AEFI data are publicly accessible via the Database of Adverse Event Notifications (DAEN), but there is a 3-month lag in publication. Australia continues to strive towards stronger multi-jurisdictional vaccine pharmacovigilance collaborations and streamlined cohesion between the jurisdictional surveillance models.

Detecting signals as an increase in AEFI frequency from previous years is best achieved by comparing risk of AEFI in the exposed (vaccinated) population. However, in this scenario, cases were predominantly adults for whom there is little available data on the vaccinated (exposed) population as the vaccine register in Australia, the Australian Childhood Immunisation Register (ACIR), is limited to vaccines administered to children aged less than 7 years [12]. Expansion of the Register in 2016 to all age groups for vaccines on the NIP or given in general practice will partially address this gap, but the register will not include vaccines administered in specialist clinics (e.g Bacillus Calmette–Guérin, travel vaccines) [13] thus underlining the importance of spontaneous or passive surveillance collating AEFI reports for all vaccines administered and from all vaccine-recipient sub-groups [14].

To determine AEFI RR, we therefore used a proxy denominator of vaccine doses distributed. This is an approximation of the exposed population as usage (and wastage) is unknown and may therefore lead to underestimation of RR. Reports of AEFI with non-NIP TIV brands were excluded from the final investigation in all years as numbers were small and vaccine doses distributed data were not readily available. Exclusion of these non-NIP TIV reports and non-specific potential allergy-related symptoms such as itchiness or undefined rash means that this summary is a conservative approach to the signal magnitude.

Non-specific AEFI such as the allergy-related reactions reported in 2015 can give rise to subjective variations in categorisation, although these would be consistent in inter-year comparisons. Brighton Collaboration definition criteria were applied for the serious AEFI of anaphylaxis [15], which showed no significant increase from previous years. The observed increased proportion of allergy-related AEFI may equally indicate a decrease in non-allergic reports; although there is no specific reason that non-allergic reporting would be depressed and no drop in the number of non-allergy-related reports was observed. Furthermore our analysis does not consider temporal co-circulation of environmental or infective allergic triggers; however, there was no indication that either parameter was in variance to previous years.

Any analysis by vaccine brand should be interpreted with caution. Vaccine brands are targeted to different vaccinee demographics and propensity to report AEFI cannot be assumed to be similar. In Victoria, Fluarix was the main brand of TIV for healthcare workers and BioCSL Fluvax the main brand of TIV used in the community with Vaxigrip used in children aged less than 5 years. It is possible that healthcare workers were more aware of SAEFVIC and likely to report an AEFI than the general population, giving rise to reporting bias for Fluarix brand. It should also be noted that low numbers limited the comparision between brands.

In 2015 there was a delay in vaccine supply and distribution due to manufacturing delays to accommodate a two-strain change in the seasonal formulation [16]. The early non-specific signal was initially hypothesised as an anomaly of timing of administration reflecting changes in uptake [10], in particular in the healthcare worker demographic where vaccination delivery was concentrated into a shorter-than-usual timeframe. However, our analysis showed that even with realignment for season commencement, the overall proportion of allergy-related AEFI was still observed to be higher than in previous years.

TIV reactogenicity has been shown to change dramatically despite a stable manufacturing process and within a single manufacturer [17-19]. The apparent increase in allergic-type AEFI reports in 2015 did not suggest that one single brand employed in the Australian-funded TIV programs was responsible, suggesting that, if real, the incorporation of one or both of the new influenza strains for 2015 season may have carried a higher allergen component in the manufacturing processes. However, these data are from a single jurisdiction and need to be confirmed by national and international data for vaccines incorporating the same strain changes before broader hypotheses can be drawn.

The recognised limitations of passive surveillance, including unquantifiable under-reporting, potential reporting biases, unascribed causality and lack of information on the exposed population, align to the benefit of multi-faceted approaches for signal detection [14,20]. The growing number of active surveillance initiatives using targeted solicited systems or interrogation of healthcare databases have the benefit of increased sample size and can also facilitate data-linkage and hypothesis-testing studies [21-23]. However, especially in the absence of a pre-specified AEFI of interest, even large-scale active surveillance systems are unlikely to consistently detect all signals. Specific target-group restrictions may also hinder detection of unanticipated signal events. For example, Australian short message service (SMS)-stimulated reactogenicity reporting surveillance systems [24] primarily target the paediatric population and so could not inform on this predominantly adult event. An increasing number of statistical signal detection methodologies have been described, but most studies demonstrate the methodological utility retrospectively and few describe the evolution of a signal detection and investigation in real time as we describe [25].

Variation in influenza vaccine strain is a regular, if not annual, occurrence depending on wild-type virus circulation. In 2016, Australia’s immunisation programme adopted quadrivalent influenza vaccines; therefore demonstrations of effective vaccine pharmacovigilance are paramount for ensuring the safety of vaccination programs. The cross-hemispheric sharing of possible signals, even if relatively minor, may aid in early alertness and stimulated monitoring to ensure vaccine pharmacovigilance is able to accurately inform the risk profile of routine immunisation programs.
